# The Power to Flirt: Power within Romantic Relationships and Its Contribution to Expressions of Extradyadic Desire

**DOI:** 10.1007/s10508-024-02997-0

**Published:** 2024-09-16

**Authors:** Gurit E. Birnbaum, Yaniv Kanat-Maymon, Kobi Zholtack, Rafael Avidan, Harry T. Reis

**Affiliations:** 1 Baruch Ivcher School of Psychology, Reichman University (IDC, Herzliya), P.O. Box 167, 46150 Herzliya, Israel; 2 Department of Psychology, University of Rochester, Rochester, NY USA

**Keywords:** Power, Infidelity, Mate value, Sexual desire

## Abstract

Power in non-romantic contexts makes people confident in their ability to attract potential partners, increasing their mating motivation and the likelihood of acting on this motivation. Four studies investigated whether perceptions of power within romantic relationships would also activate mating goals, intensifying desires for alternative partners. In Studies 1 and 2, participants underwent power manipulation and then described a sexual fantasy or evaluated photos of attractive strangers. Studies 3 and 4 used face-to-face interaction and daily experiences methods to examine the mechanisms underlying the link between power and extradyadic desires. Overall, high perceived relationship power was associated with increased interest in alternatives. Perceived relative mate value explained this association, suggesting that what determines whether power elicits extradyadic interest is not power perceptions alone but rather the feeling of having a higher mate value than one’s partner that accompanies elevated power and affects whether high-power individuals will prioritize their own needs in ways that may hurt their partners.

## Introduction

People lack power when the fulfillment of their needs is dependent on another person’s actions, and they possess power when they can control another person’s outcomes (Kelley & Thibaut, 1978; Keltner et al., 2003). Possession of power may therefore have transformative effects on people’s psychological state (Keltner et al., 2003), making them more confident, entitled, and impulsive (De Cremer & Van Dijk, 2005; Galinsky et al., 2008; Lammers et al., 2010). The self-assurance induced by power is also manifested in sexual and romantic behavior. Power, for example, renders people more confident in their ability to attract potential partners (Lammers et al., 2011), which can amplify their mating motivation (Kunstman & Maner, 2011). This heightened mating motivation is reflected in a greater readiness to think about sexual concepts (Kunstman & Maner, 2011, Study 1) and in more sexualized conduct toward subordinates (Kunstman & Maner, 2011, Study 4). Despite this connection between possession of power outside the context of close relationships and activation of mating goals, considerably less is known about whether and why experiencing power within romantic relationships increases extradyadic mating motivation.

A handful of studies have examined how power imbalances in romantic relationships (arising from unequal status and resources, for example) are manifested in couple sexual dynamics. However, methodological limitations in these studies (e.g., reliance on correlational and cross-sectional designs; Brezsnyak & Whisman, 2004; Johansen et al., 2023) engender ambiguities in understanding the influence of relationship power in the mating arena. In the present research, we used complementary methods to examine the possibility that what determines whether relationship power would motivate people to seek sexual gratification outside the relationship is not power perceptions alone. Rather, it is the sense of having a higher mate value than one’s partner that comes with elevated power (Lindová et al., 2021) and affects whether individuals will act upon their desires without having to accommodate to their partner. Specifically, we examined whether high perceived relational power would instigate interest in alternative partners and whether this process would be explained by perception of one’s mate value relative to that of the current partner (i.e., how desirable one believes themselves to be in the “mating market” compared to their current partner, based on their subjective assessment of qualities considered attractive to potential mates; Fisher et al., 2008). For the sake of brevity and clarity, we use the phrase “extradyadic interest” to refer to thoughts, desires, and behaviors directed toward individuals outside one’s primary romantic relationship.

### The Interpersonal Implications of Power Possession

Interdependence theory proposes that the source of interpersonal power, or lack thereof, is dependence, defined as the extent to which one must rely on another person to attain desired outcomes (Kelley & Thibaut, 1978). Power is thus inherently relational, such that people who possess relatively greater degrees of power have more potential to influence, change, or control another person or to resist this person’s influence attempts. As such, people with high power are likely to be self-oriented rather than other-oriented, at least in Western cultures and outside the context of close relationships (Gruenfeld et al., 2008). High-power individuals, for example, are less likely to adopt others’ perspective and read their emotional expressions accurately (e.g., Galinsky et al., 2006; Kraus et al., 2010), and are more likely to objectify others (Gruenfeld et al., 2008) and act in line with their own, rather than others’, preferences (Galinsky et al., 2003). Even when powerful individuals recognize others’ feelings, they may still prioritize their own interests (Laurin et al., 2016), showing less compassion (van Kleef et al., 2008) and willingness to help them (Lammers et al., 2012).

And yet, possession of power is not always detrimental for relationships, inasmuch as contextual factors can change the way power is enacted (Simpson et al., 2019). In contexts where people typically hold pro-relationship goals, power may encourage the fulfillment of these goals (e.g., Chen et al., 2001; Galinsky et al., 2003). Thus, when people care about others, power may instill a sense of social responsibility (Magee & Smith, 2013) and make individuals more empathic to the needs of those who have less power (e.g., Smith & Hofmann, 2016; Tost et al., 2015). This tendency may be particularly marked in intimate relationships because of the communal orientation that typically characterizes them (Clark et al., 2010; Simpson et al., 2019) and motivates people to meet their partner’s needs without expectation of direct reciprocation (Clark & Mills, 2012). Hence, high-power partners may sometimes act in ways that make low-power partners feel supported and satisfied rather than overpowered (Simpson et al., 2019). A few studies have shown that this kind of power dynamic can help sustain healthy relationships by promoting forgiveness of low-power partners' transgressions (Karremans & Smith, 2010) and encouraging communal care for low-power partners, especially in contexts where high-power partners also feel dependent on their low-power partners, such as in coparenting young children (Overall et al., 2023, Study 5).

Although power may produce some positive relational outcomes, research indicates that relationships with power differences result in worse outcomes for both partners, at least in Western cultures, which typically uphold an expectation of equal power (Simpson et al., 2019). These outcomes include lower levels of trust, happiness, commitment, security, and stability compared to romantic relationships in which partners have equal power (Schwartz & Gonalons-Pons, 2016; Stanley et al., 2017). In particular, experiencing unequal power relative to each other (rather than the absolute experience of power) may lead both high- and low-power individuals to perceive more conflict of interest, thereby reducing trust (du Plessis, 2023).

Unequal power may also damage intimacy as the low-power partners may be overly concerned about losing their relationship (the Principle of Lesser Interest; Waller, 1938) and therefore feel less safe communicating their needs and raising complaints about their partners (e.g., Samp & Solomon, 2001; Worley & Samp, 2016). In this way, power imbalances may generate dynamics in which high-power partners are less responsive to their partners’ needs, while low-power partners tend to comply with partners, inhibiting their own needs (Knudson-Martin, 2013; Mahoney & Knudson-Martin, 2009). Over time, this dynamic likely proves harmful for the low-power partner’s well-being (Mikula et al., 2012), as manifested in greater levels of anxiety, depression, and fatigue (Walsh, 1989). The resulting dissatisfaction may explain why power disparities often drive couples to seek therapy (Parker, 2009).

In all probability, the manifestations of power dynamics in the sexual arena are no less complex. Having power increases confidence and romantic approach behavior that may facilitate goal attainment (e.g., attracting mates; Gonzaga et al., 2008; Lammers et al., 2011). At the same time, the possession of power may motivate sexual behaviors that harm relationships. Specifically, research has revealed that non-relationship power may intensify sexual urges, for example, by raising the likelihood of treating others in objectifying, overly sexual, and aggressive ways (Kunstman & Maner, 2011; Zurbriggen, 2000). Moreover, possession of relatively high levels of power outside of a relationship is associated with infidelity in both men and women (Lammers et al., 2011). In romantic relationships, perceived power has been related to destructive behaviors, such as refusal to use condoms and sexual coercion (Kaura & Allen, 2004; Pulerwitz et al., 2002). Nonetheless, to our knowledge, no research has investigated how power dynamics within a romantic relationship affect expressions of sexual interest outside the primary relationship.

### The Present Research

In the present research, we proposed that high perceived relationship power fosters the belief that one’s mate value in the mating market exceeds that of the current partner. This proposition is grounded in the understanding that experiencing a sense of power over others involves lesser dependency (Kelley & Thibaut, 1978), enhances one’s self-perception (Keltner et al., 2003), and boosts one’s confidence in their desirability to others (Lammers et al., 2011). Within the context of romantic relationships, such power dynamics may lead high-power partners to believe they can offer more valuable resources than their less powerful partners and may thus be interpreted as a sign that the high-power partners possess more alternatives outside the relationship (Overall et al., 2023) and have a higher mate value (Ellis et al., 2002; Lindová et al., 2021). We proposed that this enhanced self-perception of relative mate value determines whether, when people perceive themselves to have high relationship power, they feel they can afford to express interest in alternative, potentially higher-value partners (Birnbaum, 2018; Buss et al., 2017). Supporting this reasoning, research has found that people who see themselves as having higher mate value than their partners are often less satisfied in their relationships and are viewed by their partners as more likely to be unfaithful (Buss & Shackelford, 1997; Conroy-Beam et al., 2016; Sela et al., 2017).

In four studies, we examined how temporary perceptions of relationship power influence interest in alternative partners. We hypothesized that perceiving oneself as having high relationship power would be associated with increased interest in alternative partners and that self-perceived relative mate value would mediate this association. We did not initially expect that high perceived relationship power would lead men and women to differ in their desire for alternative partners because studies typically do not find gender differences in the relationship outcomes of power (e.g., Korner & Schutz, 2021; Overall et al., 2023). Still, considering that gender differences are evident in the desire and readiness to engage in extradyadic sex (e.g., Boekhout et al., 2003), we explored the interactive effect of gender and power on expressions of extradyadic desire across all studies.

In Study 1, we sought to overcome the limitations of past correlational studies by establishing a causal connection between relationship power and the experience of extradyadic thoughts and desires. To do so, partnered participants underwent power manipulation and then described a sexual fantasy in narrative form. These fantasies were coded for expressions of sexual desire for alternative partners. In Study 2, we wished to replicate the predicted effect of relationship power on extradyadic desires while minimizing the likelihood of method-bound results. We did so by using a different measure of extradyadic desires that circumvented defenses, assessing automatic approach tendencies to attractive alternatives. Specifically, partnered participants carried out the same power manipulation as in Study 1 and then evaluated photos of strangers, indicating under time pressure whether they would consider each one of them to be a potential partner.

In Study 3, we investigated the process underlying the link between power and extradyadic desires, while focusing on whether the feeling of having a higher mate value than one’s partner might mediate this link. Partnered participants described recent events that reflected the power dynamics in their relationship and rated their perception of relationship power as well as their mate value relative to that of their partner. They then interacted face-to-face with an attractive confederate and rated their sexual desire for this confederate. In Study 4, we sought to explore whether the proposed mediation model would generalize to everyday life and be manifested in actual extradyadic behavior. For this purpose, both members of heterosexual couples completed daily measures of perceived relationship power, perceived relative mate value, and engagement in sexual activities with someone other than the current partner over three weeks. All studies were approved by the local ethics institutional review board and preregistered. In Studies 1–3, we report all measures, manipulations, and exclusions. In Study 4, which is part of a larger ongoing project without other publications to date, we report only measures relevant to the current research.

## Study 1

Study 1 was designed to establish a causal connection between perceptions of power in the current relationship and experiencing extradyadic thoughts and desires. For this purpose, romantically involved participants were asked to describe either a past situation in which they possessed power over their current partner or a typical day in their life as a couple. Participants were then instructed to write an open-ended narrative of an extradyadic sexual fantasy involving someone other than their current romantic partner. We explicitly focused on alternative partners to align with our study’s objective of examining how relationship power affects sexual thoughts and desires beyond the confines of the primary relationship. These instructions were also chosen to mitigate potential social desirability biases that might have led participants to report fantasies involving their current partners, even if they were actually fantasizing about others. Independent raters coded these fantasies for expressions of sexual desire for alternative partners. We hypothesized that participants in the power condition would incorporate greater sexual desire for alternative partners than participants in the control condition.

## Method

### Participants

A total of 128 Israeli undergraduate students (64 women, 64 men) participated in the study for course credit. Sample size was determined via a priori power analysis using G*Power software package (Faul et al., 2009) to ensure 80% power to detect an effect size, *d*, of 0.50 at *p* < 0.05. We based this hypothesized effect size on findings from past research examining the association between relationship power and sexual desire (Brezsnyak & Whisman, 2004). A sensitivity power analysis^1^ in G*Power indicated that a sample of 128 participants was sufficient to detect an effect size of *d* = 0.50 or greater in an independent-samples *t*-test of desire for alternative partners (*α* = 0.05, power = 0.80). Potential participants were recruited if they were in a monogamous mixed-sex relationship of at least 4 months duration. Participants ranged from 21 to 32 years of age (*M* = 25.10, SD = 2.26). Relationship length ranged from 4 to 192 months (*M* = 30.64, SD = 44.94).

### Measures and Procedure

Participants who wished to take part in a study of interpersonal experiences and expressions of intimacy attended a single half-hour laboratory session. Prior to each session, participants were randomly assigned to one of two conditions: relationship power and a control condition. Upon arrival at the laboratory, participants underwent a power manipulation, adapted from Galinsky et al. (2003, Study 2) to activate a sense of power within romantic relationships (Laurin et al., 2016, Study 2). Participants in the power condition were asked to provide a written description of a time when they possessed power over their partner. The experimenter explained that by power, we meant a situation in which participants controlled the ability of their partner to get something the partner wanted, such as getting to decide whose parents to spend holidays with or seeing participants’ choice of movie. Participants were instructed to describe in detail both what had happened and how they had felt about the experience at the time. Participants in the control condition were asked to describe a typical day in their lives as a couple from morning to night. In both conditions, the experimenter reviewed participants’ descriptions to ensure adherence to the instructions before moving forward.

After describing their experiences, participants completed four items assessing their perceptions of relationship power during the experience they described. These items were taken from the Sense of Power Scale (Anderson et al., 2012; “To what extent did you feel power over your partner?”; “To what extent did you feel that you got to make the decisions?”; “To what extent did you feel that you could get your partner to do what you wanted?”; and “To what extent did you have more control over the relationship than your partner and were the one managing it?”; *α* = 0.87) and were rated on a 5-point scale ranging from 1 (*not at all*) to 5 (*very much*). Upon completing these items, participants were presented with the definition of the term “sexual fantasy” (Leitenberg & Henning, 1995), as follows:

Sexual fantasies refer to any mental imagery that is sexually arousing or erotic to the individual. A sexual fantasy can be an elaborate story, or it can be a fleeting thought of some romantic or sexual activity. It can involve bizarre imagery, or it can be quite realistic. It can involve memories of past events, or it can be a completely imaginary experience.

Then, participants were given the following instructions (Birnbaum et al., 2019a):

Please think of a sexual fantasy that involves someone other than your current romantic partner. Please write about the first fantasy that comes to mind in the space below and describe in detail the specific scene, series of events, figures, wishes, sensations, feelings, and thoughts that are experienced by you and the other figures in your fantasy. At this point, we wish to note that you are writing anonymously, so feel free to write anything you like.

After writing their fantasy, participants provided basic demographic information (e.g., age and relationship length) and were fully debriefed.

*Coding Fantasmatic Expressions of Sexual Desire for Alternative Partners*. Two psychology students, who were blind to the hypotheses and experimental conditions, had been trained to code participants’ sexual fantasies. Each coder reviewed the fantasy narrative and independently assessed whether it involved individuals other than the participant’s current partner, ensuring that the fantasies indeed featured alternative partners. Coders then rated each participant’s expressions of sexual desire for the target of the fantasy in a single overall coding of sexual desire for alternative partners. The expressions of sexual desire involved articulated interest in having sex as well as explicit descriptions of foreplay (e.g., kissing, making out) and sexual intercourse. This coding scheme has been successfully used in prior research (Birnbaum, 2022; Birnbaum et al., 2019c, 2022). The coders made their ratings on a 5-point scale ranging from 1 (*not at all*) to 5 (*very much*). Inter-rater reliability was high (ICC = 0.83). Hence, coders’ ratings were averaged for each participant.

## Results and Discussion

### Manipulation Check

A *t*-test on the sense of relationship power yielded the expected effect, *t*(126) = 6.08, *p* < 0.001, Cohen’s *d* = 1.07, 95% confidence interval (CI) for Cohen’s *d* [0.70, 1.43]. Participants in the power condition experienced greater relationship power (*M* = 3*.*21, SD = 1*.*05) than did participants in the control condition (*M* = 2*.*16, SD = *0.9*0).

### Main Analyses

Descriptive statistics are presented in Table 1. To examine the effect of power manipulation on desire for alternative partners as expressed in sexual fantasies, we conducted a 2 (power) × 2 (gender) analysis of variance (ANOVA).^2^ The analysis did not yield a significant effect for the manipulation of power, *F*(1, 124) = 0.67, *p* = 0.415,* η*_*p*_^*2*^ = 0.005, 95% CI [0.00, 0.06], nor for gender, *F*(1, 124) = 0.75, *p* = 0.389,* η*_*p*_^*2*^ = 0.006, 95% CI [0.00, 0.06]. However, the analysis yielded a significant interaction between the manipulation of power and gender, *F*(1, 124) = 4.52, *p* = 0.035,* η*_*p*_^*2*^ = 0.035, 95% CI [0.01, 0.12]. Simple effects tests revealed that the manipulation of power had a significant effect on men’s desire for alternatives, *F*(1, 124) = 4.36, *p* = 0.039,* η*_*p*_^*2*^ = 0.034, 95% CI [0.01, 0.12], such that men expressed greater sexual desire for alternative partners in the power condition (*M* = 3.25, SD = 1.48) than in the control condition (*M* = 2.56, SD = 1.32). The manipulation of power had no significant effect on women’s desire for alternatives, *F*(1, 124) = 0.85, *p* = 0.358,* η*_*p*_^*2*^ = 0.007, 95% CI [0.00, 0.06], such that women’s desire for alternatives in the power condition (*M* = *2*.55, SD = 1.32) did not significantly differ from women’s desire for alternatives in the control condition (*M* = 2.85, SD = 1.14).

**Table 1:** Descriptive statistics of the measures used in Study 1

	Perceived power	Desire for alternatives
Perceived power	–	.22*
Mean (SD)	2.66 (1.10)	2.80 (1.33)

*N* 128. All items were rated on 5-point Likert scales; * *p* < .05

These findings indicated that the salience of relationship power mainly increased men’s desire for sex with alternative partners, at least in their sexual fantasies. Past research has shown that men express greater desire and willingness to engage in extradyadic sex than women (e.g., Boekhout et al., 2003). Our findings suggest that perceiving higher levels of relative power amplifies these tendencies, motivating men to express such desires in their fantasies. It is possible that men’s desire for sex with alternative partners is more readily stimulated by power than that of women, as it aligns with masculine (but not feminine) norms of demonstrating power (Bosson & Vandello, 2011; Vescio et al., 2010).

Still, it is unclear whether women’s sexual desire for alternatives is less affected by their own sense of power than that of men or whether the method used in this study accounts for these gender differences. For example, past studies have revealed that a larger proportion of men’s sexual fantasies involve someone other than their current partner, as compared to that of women (Birnbaum et al., 2019a; Hicks & Leitenberg, 2001). Men’s fantasies may be more likely to reflect this tendency upon feeling a sense of power over their partner, which could make them believe they can afford replacing their current mate with a more desirable partner (Ellis et al., 2002; Lindová et al., 2021), inspiring them to imagine this possibility. However, this does not necessarily mean that less private expressions of desire (e.g., openly expressing interest in alternative mates) are affected in the same way by such a sense of power. Another possible explanation involves the instructions for the fantasy task. Participants were asked to write specifically about alternative partners rather than about any object that came to their mind. We therefore cannot know whether the same results would have emerged if participants would have experienced extradyadic desires if not given this instruction. Study 2 addressed these limitations.

## Study 2

In Study 2, we sought to replicate the effects observed in Study 1, employing a different measure of interest in alternative partners that enabled us to examine automatic approach tendencies toward attractive alternatives (van Straaten et al., 2009). To do so, romantically involved participants went through the same manipulation of relationship power as in Study 1. Then, participants were instructed to view photos of strangers of the same gender as their partner and indicate, under time pressure, whether they would consider each of the strangers in the photos to be a potential partner. The number of selected potential partners served as an indicator of interest in alternatives. We hypothesized that participants in the power condition would express interest in a greater number of alternative partners than participants in the control condition.

## Method

### Participants

A total of 133 Israeli undergraduate students (69 women and 64 men) participated in the study for course credit. Sample size was determined via a priori power analysis using G*Power software package (Faul et al., 2009) to ensure 80% power to detect an effect size, *d*, of 0.50 at *p* < 0.05.^3^ A sensitivity power analysis in G*Power indicated that a sample of 133 participants was sufficient to detect an effect size of *d* = 0.49 or greater in an independent-samples t-test of interest in alternative partners (*α* = 0.05, power = 0.80). Potential participants were recruited if they were in a monogamous mixed-sex relationship of at least 4 months duration. Participants ranged from 20 to 40 years of age (*M* = 25.65, SD = 3.87). Relationship length ranged from 4 to 228 months (*M* = 40.42, SD = 35.49).

### Measures and Procedure

Participants who wished to take part in a study of interpersonal experiences and preferences followed the same procedure as in Study 1, including the completion of four items assessing their perceptions of relationship power during the experience they described (*α* = 0.86). The only difference was that, following the power manipulation, participants evaluated alternative partners rather than described an extradyadic fantasy. Specifically, using Ritter et al.’s (2010) procedure for assessing derogation of attractive alternatives, participants were asked to view photos of 20 attractive and 20 unattractive strangers of the same gender as their partner. The photographs, which were taken from a prior study that employed the same procedure (Birnbaum et al., 2019b; Study 2), were presented in randomized order.

For each photograph, participants were instructed to indicate, by pressing the “yes” or “no” button within 800 ms, whether they would consider this person to be a potential partner. We used this very brief time frame to ensure that participants had little opportunity to deliberately regulate their decision-making process. We summed up the number of attractive alternatives that were selected as potential partners, such that a lower value indicated lesser interest in alternatives, whereas a higher value indicated greater interest. Finally, participants completed basic demographic information and were fully debriefed.

## Results and Discussion

### Manipulation Check

A *t*-test on the sense of relationship power yielded the expected effect, *t*(131) = 3.43, *p* < 0.001, Cohen’s *d* = 0.59, 95% CI for Cohen’s *d* [0.25, 0.94]. Participants in the power condition experienced greater relationship power (*M* = 3*.*07, SD = 1*.*23) than did participants in the control condition (*M* = 2*.*41, SD = *0.9*4).

### Main Analyses

Descriptive statistics are presented in Table 2. To examine the effect of power manipulation on interest in alternative partners, we conducted a 2 (power) × 2 (gender) ANOVA. This analysis yielded a significant effect of the power manipulation on interest in alternatives, *F*(1, 129) = 4.50, *p* = 0.036,* η*_*p*_^*2*^ = 0.034, 95% CI [0.01, 0.11], such that participants in the power condition expressed greater interest in alternatives (*M* = 11.57, SD = 4.66) than did participants in the control condition (*M* = 9.85, SD = 4.80). The analysis did not yield a significant effect for gender, *F*(1, 129) = 0.53, *p* = 0.469,* η*_*p*_^*2*^ = 0.004, 95% CI [0.00, 0.05] nor for the interaction between the manipulation of power and gender (see Table 3).

**Table 2:** Descriptive statistics of the measures used in Study 2

	Perceived power	Interest in alternatives
Perceived power	–	.04
Mean (SD)	2.76 (1.14)	10.74 (4.79)

*N* 133. Items of perceived power were rated on 5-point Likert scales. The selected number of potential alternative partners, indicative of an interest in alternatives, could vary from 0 to 20

**Table 3:** Means, standard deviations, statistics, and effect sizes of interest in alternatives for the experimental conditions (Study 2)

	Power		Control		*F*(1, 129)	*η*^2^	95% CI for *η*^2^
	Men	Women	Men	Women			
Interest in alternatives	12.03 (4.64)	11.18 (4.69)	10.03 (4.52)	9.68 (5.16)	0.90	.001	[.00, .06]

*N* 133. Standard deviations are presented in parentheses. The *F*-value refers to the interaction between power and gender. The selected number of alternative partners indicates interest in them

Study 2 showed that high perceived relationship power increased both men’s and women’s likelihood of considering attractive others as potential partners. This effect was established using a measure of desire for attractive alternatives that is less sensitive to conscious control than the one employed in Study 1, as it assesses automatic approach tendencies, and thereby bypasses deliberate and thoughtful processes. Overall, the results imply that a sense of relationship power decreases the motivation to protect the relationship from the allure of alternative mates, at least as reflected in expressed interest in such mates. However, it is worth noting that the participants in Study 2 evaluated hypothetical alternative partners rather than actual individuals who could potentially be available to them (e.g., were students on campus). This raises questions about whether the interest in alternatives expressed in this study would translate to real-life situations where individuals might face a more realistic, imminent threat to their current relationship. Study 3 addressed this limitation.

## Study 3

In Study 3, we wished to replicate and extend the findings of Study 2 by exploring whether the predicted effect of relationship power on extradyadic desires would generalize to face-to-face, spontaneous interactions with an attractive stranger. Additionally, we investigated the role of perceived relative mate value as a potential mechanism underlying the link between relationship power and heightened extradyadic desires. Unlike Studies 1 and 2, in which power was manipulated experimentally, we asked romantically involved participants to describe recent events that reflected the power dynamics in their relationship and then rate their perceptions of relationship power and relative mate value. Upon completion, they completed a task in which they built a five-floor pyramid with an attractive confederate and rated their sexual desire for the confederate. We hypothesized that perceived relationship power would be associated with perceived relative mate value, which, in turn, would predict greater sexual desire for the confederate.

## Method

### Participants

A total of 130 Israeli undergraduate students (64 women, 66 men) participated in the study for course credit. Following Fritz and MacKinnon's (2007) suggestion, sample size was determined via a priori power analysis using PowMedR in R (Kenny, 2013) to provide over 90% power to detect a medium-sized effect (0.30 in a correlation metric) for both paths a and b in a mediation analysis. A sensitivity power analysis indicated that a sample of 130 participants was sufficient to detect a medium-sized effect (standardized indirect effect = 0.08; *α* = 0.05, power = 0.80). Potential participants were recruited if they were in a monogamous mixed-sex relationship of at least 4 months duration. Participants ranged from 18 to 50 years of age (*M* = 24.65, SD = 3.08). Relationship length ranged from 4 to 312 months (*M* = 37.00, SD = 41.89).

### Measures and Procedure

Participants who wished to take part in a study of interpersonal experiences attended a single half-hour laboratory session. Upon arrival at the laboratory, an experimenter greeted the participants and informed them that they were about to describe expressions of power in their relationship. The experimenter then asked participants to recall and describe recent events that reflect the power dynamics in their relationship, using an adaptation of the procedure of Laurin et al. (2016). Specifically, participants were told that over the course of romantic relationships, people tend to experience ups and downs in perceptions of their power within the relationship, such that sometimes they may control the ability of their partner to get something they want, and sometimes their partner may have power over them.

Participants then were asked to describe how power played out in their relationship by recalling specific events that illustrated the power dynamic (e.g., getting to decide whose parents to spend the holidays with) and describing how they felt. Next, participants were asked to complete the items assessing their perceptions of relationship power described in Study 1 (*α* = 0.80) and four items assessing their perception of their relative mate value, which were adapted from Birnbaum et al. (2021) to reflect the perception of participants’ mate value compared to that of their partner (“I would be perceived by other people as a more desirable mate than my partner”; “If my partner and I would break up, it would be easier for me than for my partner to find another partner for a long-term relationship”; “I perceive myself as a more valuable mate than my partner”; and “My worth in the dating market is higher than that of my partner”; *α* = 0.80). These and all other items were rated on a 5-point scale ranging from 1 (*not at all*) to 5 (*very much*).

After listening to their description, the experimenter followed the procedure used by Birnbaum et al. (2023) and informed the participants that in the next 5 min, they and another participant were about to build a five-floor pyramid, using plastic wine cups. In reality, all participants were assigned the same attractive confederate of the same gender as their partner, who was blind to the hypotheses. The experimenter then introduced the confederate to the participant and left the room. After five minutes, the experimenter returned and led the participant and the confederate into separate rooms to ensure confidentiality. Participants were then asked to complete four items assessing their desire to engage in making out and having sexual intercourse with the confederate (Birnbaum et al., 2011; “To what extent would you be interested in kissing the other participant?”; “To what extent would you be interested in fooling around with the other participant?”; “To what extent would you be interested in making out with the other participant?”; and “To what extent would you be interested in having sex with the other participant?”; *α* = 0.97). To mask their intent, these items were intermixed with four filler items (e.g., “To what extent would you be interested in going for a walk with the other participant?”). Upon completion of the questionnaire, participants were asked to provide basic demographic information and were fully debriefed.

## Results and Discussion

**Fig. 1:**
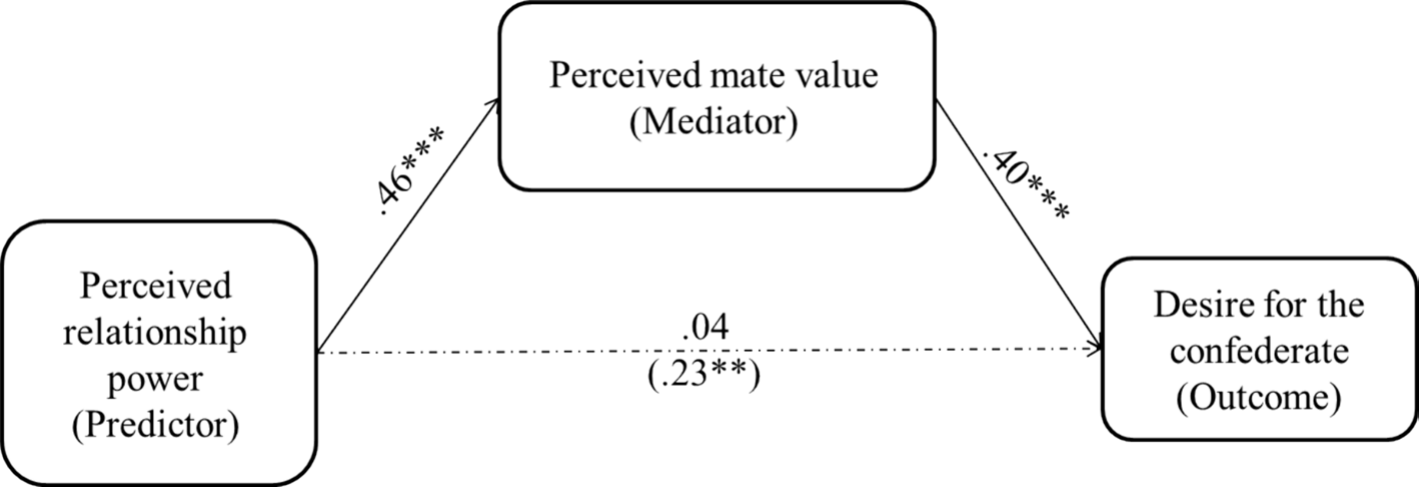
Mediation model showing that perceived mate value mediated the association between perceived relationship power and the desire for sex with someone other than the current partner (a confederate) in Study 3. Path coefficients are standardized. The value in parentheses is from the analysis of the effect without perceived mate value in the equation. ** p < .01, *** p < .001

To examine our hypothesis about mediation, we used PROCESS (Hayes, 2013, model 4), in which the perceptions of relationship power were the predictor, sexual desire for the confederate was the outcome measure, and perceived relative mate value was the mediator (Descriptive statistics are presented in Table 4). Figure 1 shows the final model. This analysis revealed a significant effect of perceived relationship power on perceived relative mate value (*b* = 0.37, *SE* = 0.06, *t* = 5.89, *p* < 0.001, *β* = 0.46, 95% CI [0.32, 0.60]), and a significant effect of perceived relative mate value on desire for the confederate (*b* = 0.57, *SE* = 0.11, *t* = 5.28, *p* < 0.001, *β* = 0.42, 95% CI [0.26, 0.58]). Also, perceived relative mate value was uniquely associated with desire for the confederate after controlling for perceived relationship power (*b* = 0.54, *SE* = 0.12, *t* = 4.45, *p* < 0.001, *β* = 0.40, 95% CI [0.22, 0.58]).

**Table 4:** Descriptive statistics of the measures used in Study 3

	Perceived power	Mate value	Desire for alternatives
Perceived power	–	.46***	.23**
Mate value		–	.42***
Mean (SD)	2.85 (1.09)	2.03 (0.88)	1.93 (1.19)

*N* 130. All items were rated on 5-point Likert scales; ** *p* < .01, *** *p* < .001

More importantly, results indicated that the 95% CI of the indirect effect for perceived relationship power as a predictor of desire for the confederate through perceived relative mate value did not include zero and thus is considered significant (*b* = 0.20, *SE* = 0.05, *β* = 0.19, 95% CI [0.09, 0.28], 5,000 bootstrapped samples). Furthermore, an alternative model, which posits that the association between perceived relative mate value and desire for the confederate is mediated by perceived relationship power, did not yield a significant indirect effect (*b* = 0.03, *SE* = 0.06, *β* = 0.02, 95% CI [-0.05, 0.11], 5,000 bootstrapped samples). These analyses supported our hypothesized mediation model, such that perceived relationship power was associated with perceived relative mate value, which, in turn, predicted greater sexual desire for someone other than the partner (a confederate).^4^

These findings not only replicated those of Study 2 but also demonstrated the utility of our mediation model. The results indicate that the sense of having higher mate value than one’s partner, which came with elevated relationship power, increased the likelihood of allowing oneself to desire alternative partners while ignoring the potentially broader implications for the current relationship (e.g., hurting partners’ feelings and endangering the relationship with them). And yet, Study 3 was cross-sectional in nature, involved only a single interaction with a single confederate, and relied on self-reports that might be affected by social desirability and motivated construal (Reis & Gable, 2000). More importantly, Study 3 was not a dyadic study and therefore did not take into account interdependence between partners and the possibility that partners’ perceptions of power may not necessarily be inversely related (Overall et al., 2023). Furthermore, because we assessed extradyadic desires rather than behavior, we could not tell whether the desire for alternative partners would translate into actual romantic or sexual advances. Finally, as in Studies 1 and 2, we did not measure sexual desire for the current partner, leaving it unclear whether relationship power specifically enhances extradyadic desire or increases desire more generally. Study 4 addressed these limitations.

## Study 4

Study 4 employed a daily experiences methodology to explore whether the findings of Study 3 would generalize to everyday life and be manifested in actual extradyadic behavior. Specifically, we asked both members of heterosexual couples to provide daily reports of perceived relationship power, perceived relative mate value, and engagement in extradyadic behavior for a period of 21 consecutive days. To control for relationship quality, we included a daily measure of relationship quality (which we failed to assess in Studies 1–3). We predicted that the association between daily perceived relationship power and daily engagement in extradyadic behavior would be mediated by daily perception of relative mate value, such that from one day to the next, participants who reported increases in perceptions of relationship power would report increases in perceptions of their relative mate value and, in turn, would be more likely to engage in extradyadic behavior.

## Method

### Participants

A total of 123 heterosexual Israeli couples participated in exchange for 300 NIS (about $90 US). Power was determined using the PinT V2.1 computer program (Bosker et al., 2003). Power for a random coefficient model was estimated for a sample of 123 couples and 21 time periods, with a moderate effect size (0.30 in a correlation metric) and yielded a power of 0.99. A sensitivity power analysis indicated that a sample of 246 participants was sufficient to detect a small-sized effect (0.06 in a correlation metric; *α* = 0.05, power = 0.80). All couples were recruited by a professional Israeli research company. Potential participants were recruited if they (a) were in a steady monogamous mixed-sex relationship of at least 4 months duration; (b) were currently sexually active. Women ranged in age from 20 to 69 years (*M* = 34.07, SD = 7.58) and men ranged in age from 20 to 71 years (*M* = 36.05, SD = 7.99). Ninety-one percent of the couples were cohabiting, and 74% were married. Seventy percent had children. Relationship length ranged from 6 to 500 months (*M* = 105.25, SD = 80.81).^5^

### Measures and Procedure

Couples who fulfilled the inclusion criteria were instructed to fill out the diary questionnaires independently and to refrain from discussing responses with their partner until the completion of the study. Emails containing a link to the daily-level measures were sent independently to both partners each day for 21 days. At the end of the study, both partners were debriefed.

*Daily-level Measures*. On each diary day, participants completed measures of perceptions of relationship power, perceptions of relative mate value, engagement in extradyadic behavior, and relationship quality. Participants also reported on their extradyadic fantasies and sexual desire for their current partner. Although these measures were included for other purposes as part of a larger project, and consequently were not included in our original hypotheses, we present the pertinent items and their associated results. All daily items were rated on a 5-point scale ranging from 1 (*not at all*) to 5 (*very much*). Given that the traditional Cronbach’s alpha is not suitable for calculating inter-item reliability in nested data, we followed guidelines suggested by Nezlek (2012) to estimate scale reliability. Specifically, in addition to the hierarchical levels of days and person, we created a third lower level that captures inter-item variability. We then ran a 3-level unconditional model in HLM 7 software (Raudenbush et al., 2011) that estimates lower-level data reliability, which is equivalent to Cronbach’s alpha.

*Perceived Relationship Power*. We used three items that were described in Study 1 and were adapted to reflect how powerful participants felt in participants’ relationships on that day (e.g., “I felt I had power over my partner today”; “Today I felt that I got to make the decisions in the relationship”; “Today, I felt that I had more control over the relationship than my partner and was the one managing it”; *α* = 0.63).

*Perceived Relative Mate Value*. Participants completed three items that were described in Study 3 and were adapted to reflect the perception of participants’ mate value relative to that of their partner on that day (e.g., “Today I would be perceived by other people as a more desirable mate than my partner”; *α* = 0.65).

*Extradyadic Fantasies*. Participants completed two items assessing the extent to which they engaged in extradyadic fantasies on that day (“Today I experienced sexual fantasies about someone other than my partner”; “Today I had romantic thoughts about someone other than my partner”; α = 0.76).

*Extradyadic Behavior*. Participants completed three items assessing the extent to which they engaged in extradyadic behavior on that day (“Today I flirted with someone other than my partner”; “Today I engaged in sexual activity with someone other than my partner”; “Today I engaged in activity with someone other than my partner that would have aroused jealousy in my partner”; *α* = 0.73).

*Sexual Desire for the Partner*. Participants completed two items assessing the extent to which they experienced sexual desire for their partner on that day (Birnbaum et al., 2016; “I was very interested in having sex with my partner today”; “I wanted to kiss my partner passionately today”; *α* = 0.69).

*Relationship Quality.* Participants rated the quality of their relationship with their partner on that day. Ratings were made on a 5-point scale, ranging from (1) “poor” to (5) “excellent.”

## Results and Discussion

To examine whether the association between daily perceived relationship power and daily engagement in extradyadic behavior would be mediated by daily perception of relative mate value, we used dynamic structural equation modeling (D-SEM), which is a recent development in the M*plus* statistical software, designed to accommodate unique features of intensive longitudinal data (Asparouhov et al., 2018). Specifically, D-SEM combines (a) time-series analysis to allow lagged relations for modeling effects between adjacent repeated measures; (b) multilevel modeling to accommodate repeated measures nested within multiple individuals and allow for individual differences in parameters; and (c) structural equation modeling for multivariate models that allows the use of latent variables and flexible structural models, enabling the inclusion of multiple predictors, mediators, or outcomes (McNeish & Hamaker, 2020).

D-SEM models are estimated with Bayesian Markov Chain Monte Carlo (MCMC) because traditional frequentist methods like maximum likelihood (ML) often encounter convergence issues or are intractable. The major difference between ML and Bayesian MCMC is that ML yields a single point estimate for each parameter, whereas MCMC yields a distribution of possible values, known as the *posterior distribution*. Similar to the frequentist point estimate and confidence interval, Bayesian MCMC provides posterior median and 95% credible interval. By providing a posterior distribution, MCMC credible intervals are more robust analogs of standard errors or confidence intervals that do not rely on assumptions or asymptotic theory. A credible interval that does not include the value 0 is equivalent to a significant result.

Similar to multilevel models, D-SEM is an inherently hierarchical model that is suitable for nested data. Yet, compared to mixed effect models, D-SEM can address challenges related to centering and effect disaggregation by incorporating latent centering from SEM that has been shown to eliminate both Ludtke’s bias and Nickell’s bias in multilevel time-series models with lagged predictors (Asparouhov et al., 2018). Moreover, the flexibility of the D-SEM allows researchers to model longitudinal dyadic data. To address the dyadic nature of repeated measures data collected from both partners, Laurenceau and Bolger (2012) expended the traditional multilevel modeling to accommodate correlated errors (nonindependence) across time and dyadic partners simultaneously. We thus applied Laurenceau and Bolger’s (2012) longitudinal dyadic modeling within the D-SEM approach.

The dyadic D-SEM analyses included three sets of control variables. First, autoregression was controlled to rule out serial dependency from one day to the next in the model variables. For example, in predicting today’s extradyadic behavior, yesterday’s extradyadic behavior was partialed out. Second, we controlled daily relationship quality to better identify the unique effects of perceived power and relative mate value over and above the general affective tone of the relationship. Lastly, as advocated by Bolger and Laurenceau (2013), we included between-person averages (e.g., latent mean self-report responses aggregated across all 21-diary days) for all primary variables (Descriptive statistics are presented in Table 5).

**Table 5:** Descriptive statistics of the measures used in Study 4

	1	2	3	4	5	6	7	8
1. Perceived power	.36***	.56***	.53***	.29***	.37***	.45***	2.89	0.86
2. Mate value	.58***	.25***	.43***	.29***	.42***	.43***	2.83	0.93
3. Extradyadic behavior	.59***	.58***	.60***	.17***	.62***	.48***	1.94	1.00
4. Relationship satisfaction	.21***	.16***	.14***	.44***	.04*	.31***	4.27	0.80
5. Extradyadic fantasies	.45***	.54***	.61***	.02	.39***	.31***	2.01	1.15
6. Desire for current partners	.24***	.22***	.22***	.32***	.10**	.20***	2.87	1.09
7. M	2.83	2.93	2.18	4.28	2.09	3.45	–	–
8. SD	0.88	0.94	1.07	0.77	1.09	1.02	–	–

*N* 246. All items were rated on 5-point Likert scales. The diagonal displays the correlations between male and female partners. Above the diagonal, the correlations pertain to female partners, whereas below the diagonal, the correlations pertain to male partners. *** *p* < .001

**Fig. 2:**
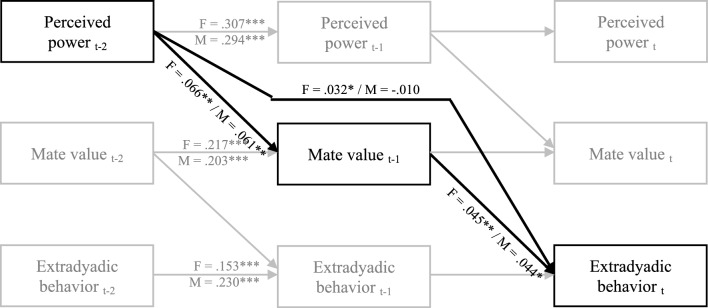
Path diagram for daily longitudinal cross-lagged effects of perceived power on extradyadic behavior via mate value (Study 4). *F* female, *M* male; Values are daily (within level) posterior medians. Relationship quality, error terms, intercepts, and dyadic covariance terms are not presented to make the figure more legible. * *p* < .05, ** *p* < .01, *** *p* < .001

To align with recommendations on longitudinal mediation models (Goldsmith et al., 2018), we applied lagged predictions of both the mediator and outcome (see Fig. 2). Thus, in each day, the mediator perceived relative mate value was predicted by yesterday’s perceived power (*a*-path), controlling for yesterday’s perceived relative mate value and relationship quality. Each day’s extradyadic behavior was predicted by yesterday’s perceived relative mate value (*b*-path) and the perceived power of the day before yesterday (*c*’-path), controlling for extradyadic behavior and relationship quality from the previous day.

As presented in Table 6 and summarized in Fig. 2, for both male and female partners, perceived power predicted the following day’s perceived relative mate value. Mate value, in turn, predicted extradyadic behavior on the following day, such that when people felt more powerful in their relationships, they perceived their mate value as higher than that of their partner on the following day. These perceptions of relative mate value, in turn, predicted greater engagement in extradyadic behavior the next day. The indirect effect of perceived power on extradyadic behavior via perceived relative mate value was significant for both male and female partners. Similar results were found at the between levels. Thus, across the 21-day period, people who, on average, felt more powerful in the relationship tended to perceive their mate value as higher and engage in extradyadic behavior. This indirect effect was significant for both partners.

**Table 6:** The effects of power on extradyadic behavior via mate value: A dyadic D-SEM model (Study 4)

	Mediation notation	Female				Male			
		Estimate	SD	*p* value	95% CI	Estimate	SD	*p* value	95% CI
*Within*									
		Mediator: mate value							
Power t-1	a	.066	.025	.004	[.018, .116]	.061	.023	.005	[.016, .160]
Quality t-1		− .032	.023	.080	[− .078, .012]	.021	.024	.185	[− .026, .067]
		Outcome: extradyadic behavior							
Mate value t-1	b	.045	.018	.006	[.010, .080]	.044	.020	.015	[.005, .084]
Power t-2	c’	.032	.018	.034	[− .002, .067]	− .010	.020	.303	[− .050, .031]
Quality t-1		− .002	.018	.460	[− .037, .034]	− .012	.022	.282	[− .056, .030]
		Autoregression paths							
Power		.307	.020	< .001	[.267, .347]	.294	.020	< .001	[.255, .335]
Mate value		.217	.022	< .001	[.173, .260]	.203	.022	< .001	[.161, .246]
Extradyadic behavior		.153	.023	< .001	[.109, .198]	.230	.024	< .001	[.184, .277]
		Mediation							
Indirect effect		.003	.002	.010	[.000, .007]	.002	.002	.020	[.000, 007]
*Between*		Mediator: mate value							
Power	a	.694	.077	< .001	[.540, .849]	.747	.074	< .001	[.602, .893]
Quality		.165	.089	.036	[− .016, .337]	-.004	.088	.486	[− .174, .169]
		Outcome: extradyadic behavior							
Mate value	b	.316	.118	.003	[.093, .554]	.378	.094	< .001	[.193, .566]
Power	c’	.378	.132	.002	[.123, .641]	.393	.105	< .001	[.194, .597]
Quality		.038	.104	.355	[− .165, .245]	.046	.089	.305	[− .126, .222]
		Mediation							
Indirect effect		.218	.085	.003	[.064, .398]	.279	.076	< .001	[.140, .438]

Estimate is posterior median, CI is credible interval

We further examined two alternative mediation models (see supplemental material). The first was that perceived relative mate value predicted extradyadic behavior via perceived relationship power; the second was that perceived relationship power predicted perceived relative mate value via extradyadic behavior. To do so, we added these effects to the hypothesized D-SEM equations. Results failed to support these alternative mediation models. Perceived relative mate value significantly predicted perceived power (Female: *b* = 0.078, *p* < 0.001, 95%CI 0.038, 0.116; Male: *b* = 0.066, *p* < 0.001, 95%CI 0.027, 0.105), yet perceived power did not predict extradyadic behavior (Female: *b* = 0.017, *p* = 0.214, 95%CI − 0.026, 0.060; Male: *b* = 0.016, *p* = 0.256, 95%CI − 0.032, 0.064). Regarding the second alternative model, as mentioned, perceived power did not predict extradyadic behavior, and extradyadic behavior did not predict perceived relative mate value (Female: *b* = 0.011, *p* = 0.354, 95%CI − 0.044, 0.067; Male: *b* = 0.039, *p* = 0.064, 95%CI − 0.011, 0.088). Paths of the hypothesized model remained significant for both partners.

**Fig. 3:**
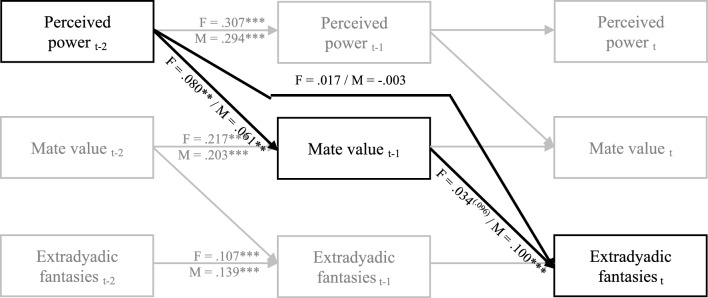
Path diagram for daily longitudinal cross-lagged effects of perceived power on extradyadic fantasies via mate value (Study 4). *F* female, *M* male; Values are daily (within level) posterior medians. Relationship quality, error terms, intercepts, and dyadic covariance terms are not presented to make the figure more legible. ** *p* < .01, *** *p* < .001

We also conducted dyadic D-SEM analyses, exploring whether the association between daily perceived relationship power and daily engagement in extradyadic fantasies would be mediated by the daily perception of relative mate value. As illustrated in Fig. 3, these analyses showed that the indirect effect of perceived power on extradyadic fantasies via perceived relative mate value was significant for male partners and marginally significant for female partners, which is somewhat consistent with the findings from Study 1. Finally, while it was not an initial focus of our research, we performed additional dyadic D-SEM analyses to explore whether a sense of power increased participants’ desire more generally, regardless of dyadic or extradyadic context. The results, presented in Table 7, revealed that perceived relationship power predicted both male and female participants’ sexual desire for the current partner the following day. These findings add to earlier research, which found that power is associated with agency and romantic pursuit (Lammers et al., 2011; Rucker et al., 2018), indicating that the influence of power on sexual desire extends beyond just extradyadic expressions, enhancing the overall drive for mating.

**Table 7:** The effects of power on sexual desire for the current partner: A dyadic D-SEM model (Study 4)

	Female partners				Male partners			
	Estimate	SD	*p* value	95% CI	Estimate	SD	*p* value	95% CI
*Within*								
Power t → Desire t + 1	.053	.027	.026	[.000, .105]	.062	.029	< .014	[.007, .120]
Desire t → Desire t + 1	.219	.023	< .001	[.175, .264]	.185	.023	< .001	[.140, .231]
*Between*								
Power → Desire	.718	.102	< .001	[.552, .919]	.204	.100	.021	[.009, .402]

Estimate is posterior median, CI is credible interval

Taken as a whole, Study 4 replicated the findings of Study 3 in a more natural research setting, which adds ecological validity to our results. In particular, the findings suggest that when individuals perceive themselves as having high relationship power, they tend to feel that their mate value is higher than that of their partner. This perception, in turn, may motivate them to dismiss relationship commitment and act on their desires should opportunities for short-term temptations or for switching to more beneficial mates present themselves.

However, several limitations affect these conclusions. Specifically, while relationship power was directly associated with sexual desire for the current partner, its association with desire for others was only indirect, mediated by higher perceived mate value. Future research should explore whether a sense of entitlement, the belief in finding a better partner, the feasibility of extradyadic sex, disrespect for current partners, or some other unmeasured belief underlie the effect of perceived relative mate value on engagement in extradyadic sex. Additionally, Study 4 revealed a negatively skewed distribution of extradyadic behavior, with many participants rarely engaging in such activities, while a smaller subset does so more frequently. Although our analytical approach performs robustly with asymmetric distributions (McNeish & Hamaker, 2020), this pattern complicates the conclusion that power increases the likelihood of such behavior, as it implies the relationship between power and extradyadic behaviors may not be straightforward or universal across all couples. Future research is needed to explore individual characteristics that may influence the strength of these associations.

## General Discussion

In Western cultures, most people enter romantic relationships expecting equality (Eaton & Rose, 2011; Sprecher & Felmlee, 1997). Only about half of romantic relationships, however, have a relatively equal power balance (Sprecher et al., 2006). Past research examining the relational implications of power differentials has indicated that although higher levels of power may have some positive outcomes (e.g., encouraging forgiveness of low-power partners and smoothing things over with them during conflict; Karremans & Smith, 2010; Overall et al., 2023), romantic relationships in which partners have equal power usually fare better than relationships with power differences (Schwartz & Gonalons-Pons, 2016; Stanley et al., 2017). The present research adds to these studies, demonstrating how high perceived relationship power can disrupt the intimate sphere, unleashing a desire for alternative partners.

Across four studies, we found that perceptions of power within a relationship significantly predicted a person’s inclination toward alternative partners, encompassing thoughts, desires, and actual interactions. We also showed that the sense of having a higher mate value than one’s partner that power instilled helped explain this power-extradyadic desire linkage. In Studies 1 and 2, we established a causal connection between high perceived relationship power and experiencing sexual desire for alternative partners, as manifested in both sexual fantasies and automatic approach tendencies toward attractive alternatives. In Study 3, we replicated these findings in face-to-face interactions with an attractive stranger and extended them, revealing that relationship power was associated with perceiving oneself as having a higher mate value than one’s partner, which, in turn, predicted heightened desire for the stranger. In Study 4, we showed that the findings of Study 3 generalized to everyday life, even when controlling for relationship quality, and were manifested not only in heightened desire for alternative partners but also in actually engaging in sexuality-related interactions with them. Of importance, the link between power and sexual desire for others was not direct but rather mediated through the perception of relative mate value.

Possession of power induces confidence, approach tendencies, and the perception that one is sexually desirable by others (Kunstman & Maner, 2011; Lammers et al., 2011) and is therefore likely to enhance mating motivation (Kunstman & Maner, 2011). Power also promotes a sense of self-orientation (Gruenfeld et al., 2008) and entitlement (De Cremer & Van Dijk, 2005) that may facilitate acting on this heightened mating motivation while giving less consideration to the desires of others. Past studies have demonstrated that the enhanced mating motivation induced by power may be expressed destructively either toward non-intimates when power is possessed outside the context of romantic relationships (e.g., making aggressive sexual advances; Kunstman & Maner, 2011; Zurbriggen, 2000) or toward romantic partners when power is possessed within the context of romantic relationships. For example, power imbalances often spill over into the bedroom, leading to sexual harassment and abuse of current partners (e.g., Kaura & Allen, 2004; Pulerwitz et al., 2002). The present studies are the first to reveal that power in romantic relationships affects sexual interest outside the relationship. By doing so, our findings add to prior work, indicating that perceptions of relationship power may undermine relationship well-being not only by encouraging aggressive sexual behavior within the relationship but also by pursuing sexual gratification outside of it.

The heightened sexual motivation induced by power (e.g., Kunstman & Maner, 2011) does not necessarily have adverse interpersonal consequences. For example, possession of power may instigate the self-assurance needed to initiate consensual intimate connections (Gonzaga et al., 2008; Lammers et al., 2011). Our findings suggest that within romantic relationships, this motivation potentially can become destructive. Although the relational implications were not tested directly in this research, it is possible that when individuals feel powerful and thus perceive themselves as having more relationship alternatives than their primary partners, they may be inclined to pay some sexual attention to these potentially promising alternatives. In such cases, as indicated by past research, the belief in having alternative mating options may weaken their commitment to their current relationship (Lee & O’Sullivan, 2019). The ensuing reduced need for relationship protection (Parker et al., 2022) should allow these people to prioritize their own needs in ways that may hurt their partners and undermine their relationship with them.

These findings align with the agentic–communal model of power (Rucker et al., 2012, 2018) that proposes that possession of power promotes an agentic rather than a communal orientation, freeing people from having to attend to others’ needs. Our findings suggest that within romantic relationships, this transformation of orientation may lead people to disengage from certain relationship-sustaining processes, such as commitment to their current partner (though not their desire for them), if they believe that they have more mating opportunities than their partners. This belief may allow them to feel they can either afford to lose them or replace them with a higher-value mate (Buss et al., 2017). When the motivation to protect the relationship diminishes, the likelihood of unleashing extradyadic desires upon encountering alternative partners increases (Birnbaum et al., 2019b; Lydon & Karremans, 2015).

Overall, our research advances understanding of how power is manifested in relationship dynamics, revealing that what determines whether power elicits extradyadic interest is not power perceptions alone but rather the sense of having a higher mate value than one’s partner that accompanies elevated power (Lindová et al., 2021) and influences the likelihood that individuals will succumb to the temptation of attractive alternatives rather than attend only to their partners for sexual fulfillment. These conclusions, however, should be taken with caution, as our data do not conclusively establish perceived relative mate value as a mediator rather than a confounder. The potential confounding arises because perceived relative mate value may independently shape individuals’ perception of their own power while also influencing their attraction to alternative partners, irrespective of their actual power. Therefore, the association observed in our studies between power and interest in others could be attributed to the underlying self-perception of relative mate value rather than power itself making alternatives seem more appealing. This is a distinction our studies do not resolve and should be addressed in future research.

Also, in all four studies, we concentrated solely on situational power without considering the enduring imbalance of power within a relationship or general relational power. It is plausible that situational power may have different effects than the overarching power dynamics within a relationship, a distinction that should be explored in future studies. For example, people who chronically experience low power in their relationships may exploit instances of sudden, temporary increases in power to assert dominance, which can manifest in negative behaviors such as sexual harassment (e.g., Williams et al., 2017). Furthermore, at least in Studies 1–3, we treated power as a within-dyad or between-person construct, focusing on how participants feel about their own power compared to their partner. Nevertheless, power may also be viewed as a between-dyad construct. Some participants, for example, may be involved in what is commonly referred to as a “power couple,” where both partners experience a sense of empowerment and collaborate to prosper as a unit. This dynamic may lead to greater relationship happiness compared to relationships that have an unequal power balance or an equal but relatively low power balance, where neither partner feels particularly empowered (Korner & Schutz, 2024).

Relatedly, because we did not assess communal strength, the data cannot speak to whether communal strength may attenuate the destructive manifestations of power. Existing literature suggests that high-power individuals often prioritize their own goals (e.g., Keltner et al., 2003; Rucker et al., 2018). Paradoxically, within the context of romantic relationships, power may motivate individuals who generally prioritize relationship goals (but not those who prioritize agentic goals) to further prioritize their relationship-oriented goals and focus on what is best for their partner or their relationship rather than on what is best for themselves (Simpson et al., 2019). Such a dynamic is likely more evident in established couples, particularly those parenting together, where a sense of power may be more inclined to activate communal goals (Overall et al., 2023, Study 5). This can explain why, in Study 4, which specifically included longer-term couples, power did not show a direct effect on behaviors that could harm the relationship, such as infidelity. Future studies should examine the effectiveness of our experimental approach and the possible moderating role of communal strength while assessing the distinct effects of actor and partner power on sexual expressions inside and outside longer-term relationships.

Another constraint is the potential lack of generalizability of our findings to contexts that do not assume equality, such as societies where women have little or no power. Additionally, while our work sheds light on power dynamics within traditional monogamous relationships, the translation of these dynamics to non-monogamous relationship structures remains uncertain. Moreover, exploring the degree to which partners agree in their perception of power is crucial to investigating whether it is simply a perceptual bias that predisposes individuals to experience extradyadic desires. Finally, the relatively short-term nature of our research design limits our ability to explore whether the patterns we identified contribute to long-term relationship deterioration. Future research should address these limitations by examining power dynamics in contexts with varying levels of gender equality and among those engaged in consensual nonmonogamy, while employing longitudinal designs to gain a comprehensive understanding of the long-term relational implications of power dynamics.

Notwithstanding these limitations, our research elucidates the implications of power dynamics for relationship well-being by demonstrating how and why power may make people more prone to infidelity. Because some degree of power disparity in a relationship is almost inevitable, understanding the contribution of constructive and destructive manifestations of power to the sexual arena is of both theoretical and applied significance. Our findings highlight the need for further investigation into interventions that address power imbalances in relationships, with the aim of intensifying the emotional bond between partners and promoting more constructive expressions of personal power (Magee & Smith, 2013; Simpson et al., 2019). Such interventions should strive to equalize perceptions of relative power, ensuring that each partner feels they have equal influence and value in the relationship.

## Footnotes

1 Sensitivity analyses for all studies were conducted post-data collection.

2 In Studies 1 and 2, we examined the interactive effect of gender and manipulation of power on extradyadic desires. It is important to note, though, that because these studies were not designed to detect moderation by gender, they had insufficient power to do so. Future research should consider the possibility of moderation of these effects by gender.

3 We did not design Study 2 with the necessary statistical power to detect the power-by-gender interaction observed in Study 1, because Study 2 was conducted before Study 1 had been analyzed.

4 In Study 3, we also used PROCESS (Hayes, 2013, model 7) to test a moderated mediation model with gender as a moderator of the association between power and perceived relative mate value. The index of moderated mediation was not significant, *b* = -0.35, 95% CI [-.21, .09], providing no evidence for moderated mediation.

5 Some participants were older than the pre-registered age requirement of being younger than 45 years.

## Declarations

### Data Availability

Data, codes, and supplemental materials of all studies are available at https://osf.io/4bfz8/?view_only=5c2c0d7c27034ae887ca5e16e8e89669. The preregistration for Studies 1–4 is available at https://osf.io/anmz3/?view_only=37d6168ac15f4122b0dd1b64a9fc003b; https://osf.io/m2y63/?view_only=065ddff72fad41ab84de44fa95a9c1ce; https://osf.io/9kc67/?view_only=525c1202cdf8421e8342e5bbb65ac295; https://osf.io/32fxd/?view_only=fd96936e1ff540788de92d864ddfc10b.
